# Qualitative and Quantitative Sex-Related Differences in the Perception of Single Molecules from Coffee Headspace

**DOI:** 10.3390/foods13203239

**Published:** 2024-10-11

**Authors:** Giorgia Sollai, Paolo Solari, Roberto Crnjar

**Affiliations:** Department of Biomedical Sciences, University of Cagliari, 09042 Monserrato, CA, Italy; solari@unica.it (P.S.); crnjar@unica.it (R.C.)

**Keywords:** smell, GC-O technique, VARUs intensity, gender, individual variability

## Abstract

One of the still-debated topics regarding the olfactory function concerns the presence or absence of sex-related differences in individuals. In this study, we checked for a relationship between the olfactory function of females and males and their ability to perceive single molecules, and researched how this can influence the intensity with which the complex odor formed by a set of single molecules is perceived. First, females and males were classified as normosmic or hyposmic based on the TDI olfactory score obtained using the Sniffin’ Sticks test. Subsequently, the headspace of roasted coffee beans, as a complex olfactory stimulus, was broken down into single molecules by means of a chromatographic column; these were simultaneously conveyed to a mass spectrometer (for their subsequent classification) and to the human nose, which acts as a chemical sensor by means of an olfactometer port. The results obtained with this gas chromatography–olfactometry approach show both qualitative and quantitative differences between females and males, with females performing better than males. In addition, the odor intensity reported by females when sniffing pen #10, containing coffee aroma, is significantly higher than that reported by males. In conclusion, these data highlight that the human ability to perceive both single compounds and complex odors is strongly conditioned, not only by the olfactory function of individuals, but also by their sex.

## 1. Introduction

The information coming from the external environment, which is captured and conveyed by the olfactory system towards the higher brain centers, plays an important role for all living organisms. The functions of the sense of smell can be grouped into three broad categories, as follows: alertness for environmental dangers (smoke, gas, toxic and/or harmful substances, presence of predators), influence on social relationships (mother–child recognition, selection of a partner for mating) and conditioning of eating behavior (it contributes to the location and choice of foods, both in qualitative and quantitative terms) [1,2,3,4,5,6,7,8,9,10,11,12]. In particular, individuals with smell disorders tend to isolate themselves socially, are subject to a greater number of household incidents and report preferring foods with high energy content, such as fats and sugars, adding flavor enhancers such as salt and spices, to the detriment of foods such as fruits and vegetables [13,14,15,16,17,18,19,20]. The choice of tastier, but also more caloric foods seems to compensate for the reduced gratification during a meal due to reduced olfactory stimulation. Additionally, these individuals tend to reach sensory satiety late, resulting in increased meal duration and intake of highly caloric foods [21,22,23,24,25,26,27].

Food and drink odors are generally made up of a mixture of molecules, and only some of them are sensorially relevant and considered odor-active compounds [21,22,23,24,25,26,27,28,29,30,31,32,33]. Therefore, it is important to understand which and how many molecules are sensorially active within a complex mixture, and how these can influence the intensity with which the smell of food and drinks is perceived. The coupled technique of gas chromatography–olfactometry (GC-O) allows for the separation of the single molecules that make up a mixture and, at the same time, allows for the use of the human nose as their sensory evaluator [34,35,36,37,38].

We have previously found that the ability to perceive the single molecules that make up a complex mixture, as they are eluted from the chromatographic column and conveyed to an evaluator via an olfactometric port, is directly related to the olfactory function of individuals. In fact, the higher the olfactory score obtained by each participant during the olfactory tests, the higher the number of molecules perceived [39,40,41]. As it is commonly accepted, on the basis of the scores obtained during olfactory tests, individuals can be classified as normosmic (normal olfactory function), hyposmic (reduced olfactory function) or functionally anosmic (general or specific inability to perceive odors) [3,19,42,43,44,45]. The reasons for this variability are multiple and can be of an environmental/behavioral nature (e.g., habitual smokers, sedentary lifestyle, polluted habitats), genetic (expression and functionality of OBPs, ORs, Kv1.3 channels, etc.) and physiological (age and sex) [40,42,46,47,48,49,50,51,52,53,54,55,56,57,58,59,60,61,62,63].

Based on these considerations, the main objective of this study was to evaluate the presence of any sex-related differences in the ability of individuals to perceive the single molecules that make up the complex odor of coffee, as they are eluted from the chromatographic column, both in qualitative (type of molecules smelled) and quantitative (number of molecules smelled) terms. In fact, one of the topics still under debate is related to the presence of differences in the olfactory function between females and males. Several studies have shown that females tend to perform better than males in their olfactory abilities, but none of them evaluated differences related to the ability to perceive single molecules [52,63,64,65]. This is of particular importance when considering that it has been suggested that odor-active compounds are those which contribute strongly to the aroma of the mixture [33,66]. The second objective was to confirm the relationship between olfactory function and the ability to perceive the single molecules as they are eluted from the chromatographic column. Finally, we assessed whether any differences in the number of odor-active compounds could also determine differences in the intensity with which females and males reported perceiving the odor of coffee contained in pen #10 of the identification test (one of the subtests of the Sniffin’ Sticks battery).

## 2. Materials and Methods

### 2.1. Subjects

The sixty-seven Caucasian volunteers who participated in this study (34 F, 33 M; age 29.19 ± 2.59 y; BMI 18.5–24.99 kg/m^2^) were recruited in the metropolitan area of Cagliari (Sardinia, Italy). Healthy, non-smoking subjects who reported having a good sense of smell, familiarity with the aroma of coffee and with a history of COVID-19 infection that ended at least 12 months before were included in the study. The exclusion criteria were as follows: presence of chronic pathologies such as metabolic (diabetes, obesity, dysglycemia, dyslipidemia, metabolic syndrome, circulating levels of peptide), inflammatory/autoimmune (inflammatory bowel diseases, Sjögren’s syndrome, rheumatoid arthritis, psoriasis, myasthenia gravis) and neurodegenerative disorders (Parkinson’s disease, Alzheimer’s disease, autism, mild cognitive impairment) [67,68,69,70,71,72,73,74,75,76,77,78,79,80,81,82,83,84,85,86,87,88,89].

Evaluation of the individual ability to perceive single molecules during the gas chromatography–olfactometry experiments was carried out using the detection frequency method. This method has the following two advantages: it does not require qualified evaluators and the results obtained are representative of interindividual variability [90,91,92,93,94].

On the day of the experiment, all participants had fasted for at least 90 min prior to testing and wore no perfume. Before starting the olfactory tests, each volunteer was read the experimental protocol that was previously approved by the local Ethics Committee and was asked to sign an informed consent form (Prot. PG/2018/22 of 2 January 2018).

### 2.2. Olfactory Sensitivity Screening

The TDI olfactory score, given by the sum of the scores obtained using the tests of threshold (T-test; score 0–16, obtained as the average of the last 4 reversals of 7), discrimination (D-test; score 0–16, given by the number of correct discriminations) and identification (I-test; score 0–16, given by the number of correct identifications), was used to evaluate the olfactory function of each individual. T-test, D-test and I-test represent the three subtests of the Sniffin’ Sticks test (Burghart Instruments, Wedel, Germany), based on odor-containing felt-tip pens, internationally recognized and widely used for olfactory screening [95] (for details, visit: https://www.uniklinikum-dresden.de/de/das-klinikum/kliniken-polikliniken-institute/hno/forschung/interdisziplinaeres-zentrum-fuer-riechen-und-schmecken/neuigkeiten/downloads). The reference values reported by Hummel et al. [96] were used to classify each participant as normosmic, hyposmic or functionally anosmic, based on the TDI olfactory score obtained and according to sex and age.

During the I-test, each participant also had to give their own assessment of the intensity with which the smell of coffee contained in pen #10 was perceived (from here on referred to as the coffee-odor pen), marking a sign on the “Visual Analogue Rating Units” scale (VARUs; ranging from 0–20) [97].

### 2.3. Dynamic Headspace Sampling

The volatiles were collected by means of the dynamic headspace method [37,98]. The headspace method is considered the most appropriate to obtain, in terms of volatiles, an extract whose composition is closely linked to the quality of the scent assessed by the consumer [99]. Additionally, it allows us to obtain both extracts for mass spectrometry–gas chromatography (MS-GC) and a sensory evaluation by a human subject, by means of GC-O analysis [37].

About 100 g of roasted coffee beans were placed inside a 0.5 L airtight glass vessel with a flow-through mechanism [39]. The air impregnated with the volatiles was then conveyed towards a glass tube (5 mm Ø) inserted in the collection port at the top of the vessel, containing a Porapak Q filter (150/75 mg, 50/80; Supelco, Bellefonte, PA, USA). By flushing the system with purified air for three hours at a rate of 30 L/h (500 mL/min), volatiles were recovered at room temperature. Using 1.5 mL of 1-hexane, trapped volatiles were released from the Porapak Q tube, resulting in a solution containing the isolated volatile chemicals. Samples were then stored at −20 °C until used. To verify the effectiveness of the extract obtained and the reproducibility of the chromatogram, three GC runs were conducted 24 h after sample preparation. The obtained chemical profile was identical to that obtained in the previous study [39] and comparable to that of other studies, providing evidence of its validity [100,101,102,103,104,105,106,107,108]. Before each experimental section, a GC analysis was also performed to verify that the sample was not altered.

### 2.4. Mass Spectrometry/Gas Chromatography–Olfactometry (MS/GC-O) Analysis

To perform the GC-O analyses, 1 µL of coffee extract volume was injected in the HP-INNOWax column (30 m × 0.25 mm × 0.50 µm; Agilent 19091N-233; Agilent technologies, Santa Clara, CA, USA) of the gas chromatograph (GC; Agilent 6890N). The injection volume, conveyed by a constant flow of 1.2 mL/min of He (carrier gas), was split 1:1 between the olfactometry detection port (Gerstel ODP3; Mülheim an der Ruhr, Germany) and the mass spectrometer (MS) detector (Agilent 5973; Santa Clara, CA, USA) coupled to the GC [41].

The injector temperature was set at 250 °C and the MS interface temperature was set at 260 °C. The oven temperature was maintained at 40 °C (0.2 min), 40 °C/min to 90 °C (0.50 min), 2 °C/min to 150 °C and 30 °C/min to 230 °C (12 min). The injector mode was splitless; 230 °C and 150 °C were, respectively, the temperatures for the ion source and the quadrupole mass filter. Chromatograms were recorded by monitoring the total ion current in a 40–550 mass range. The transfer line of the GC-ODP3 sniffing port was held at 220 °C [41].

The mass spectrum found in the MS Standard Library NIST2014 (US National Institute of Standards and Technology; Gaithersburg, MD, USA) was used to identify the volatiles obtained from the roasted coffee beans by means of the dynamic headspace method [39]. As previously reported [39], we found 50 different volatiles, and the information regarding “odor type” (i.e., roasted, bready, nutty, etc.) and “odor descriptors” (i.e., coffee, spicy, cheese, wood, etc.) were obtained from the Good Scents Company Information System (www.thegoodscentscompany.com), according to Gonzales-Kristeller and co-workers [109].

Every time the participant detected an odor, they had to record it on a PC, using a digital recording and reporting system (GERSTEL ODP 3 for Windows 7), as well as their own subjective evaluation of the odor-active compound perceived, such as the following: intensity, duration, hedonic value and identification [37,38]. The participants recorded their evaluation by pressing one of the 4 keys present in the reporting system, which also provided information on the perceived intensity on a 1–4 scale, as follows: 1 = weak odor, 2 = distinct odor, 3 = intense odor, 4 = very intense smell. The aromagrams were superimposed on the chromatograms by means of automatic recording of the retention and sniffing times of each odor-active compound. To avoid preconditioning, the samples were presented blindly.

### 2.5. Statistical Analysis

Fisher’s Exact Test was used to analyze differences in the perception of some odor-active compounds between females and males.

The Pearson’s correlation test was applied to examine the relationship between the following: (a) the TDI olfactory score and the total number of odor-active compounds (hereafter, total-molecules) or the number of odor-active compounds smelling of coffee (hereafter, coffee-molecules), smelled by each subject, considering females and males both together and separately; (b) the TDI olfactory score and the intensity perceived by each subject for the pen of the identification test containing the coffee aroma (hereafter, coffee-odor pen), considering females and males both together and separately; (c) the intensity perceived for the coffee-odor pen and the number of total- and coffee-molecules smelled by each subject, considering females and males both together and separately. Statistical analyses were performed using GraphPad Prism 6 (GraphPad Software, San Diego, CA, USA). A statistically significant correlation was defined with a *p*-value < 0.05.

One-way ANOVA was used to evaluate the effect of the TDI olfactory status of the subjects on their ability to smell single molecules during the GC-O tests and on the intensity perceived for the coffee-odor pen.

Two-way ANOVA was used to test for a significant interaction between TDI olfactory status × sex on the ability to detect individual molecules, both in the case of total- and coffee-molecules.

Fisher’s test of least significant difference (LSD) was used for post hoc comparisons. Statistical analyses were performed using STATISTICA for WINDOWS (version 7.0; StatSoft Inc., Tulsa, OK, USA). *p* values < 0.05 were considered significant.

## 3. Results

Table 1 shows that 48 of the 50 compounds found in the headspace of roasted coffee beans were odor-active for at least two of the participants; in fact, the “ethylbenzene” (signed as n. 5 in Table 1) was active for just one individual, while the “2-Butanone, 1-(acetyloxy)” (signed as n. 33 in Table 1) was not perceived by any participant. Furthermore, panelists perceived 23 of the 48 odor-active compounds as smelling of coffee, indicated by numbers 3, 8, 11–12, 14–16, 20–21, 23–24, 26–28, 30, 34, 37, 39–42 and 49–50 in Table 1. However, only the odor-active compounds written in red in Table 1 are defined in the literature as coffee odorants. This means that participants correctly identified 18 of the 21 molecules as smelling of coffee.

Table 2 shows the distribution of females and males in relation to their ability to perceive some odor-active compounds as they are eluted from the chromatographic column. In particular, a greater number of females than males were able to perceive the following volatiles: toluene (χ^2^ = 4.229, *p* = 0.039), pyridine (χ^2^ = 9.205, *p* = 0.003), furfural (χ^2^ = 5.230, *p* = 0.022), 2-furanmethanol acetate (χ^2^ = 5.380, *p* = 0.020), furan,2,2-methylenebis- (χ^2^ = 5.380, *p* = 0.020) and 2-furanmethanol (χ^2^ = 5.380, *p* = 0.020), belonging to coffee-molecules, and pyrazine 2-methyl-6-(2-propenyl) (χ^2^ = 6.052, *p* = 0.014) and 2-acetylpyrrole (χ^2^ = 5.490, *p* = 0.019), belonging to total-molecules. Instead, a greater number of males than females were able to perceive the following volatiles: D-limonene (χ^2^ = 4.16, *p* = 0.041), 2-propanone 1-hydroxy (χ^2^ = 4.695, *p* = 0.030) and pyrazine 3,5-diethyl-2-methyl- (χ^2^ = 5.380, *p* = 0.020), all belonging to total-molecules. No other differences in the perception of volatiles during GC-O experiments between females and males were found.

**Figure 1:**
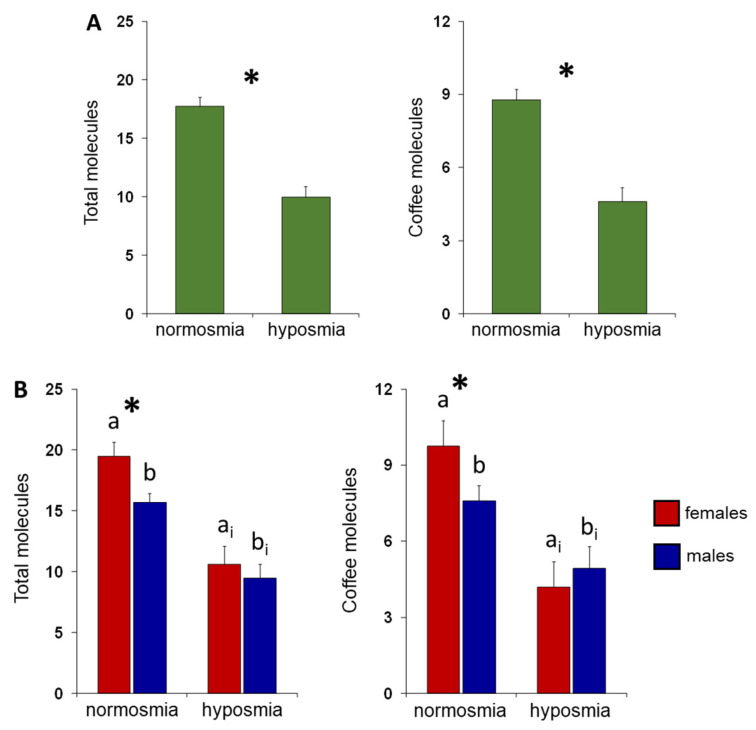
Effect of the TDI olfactory status on the ability to perceive single molecules. (**A**) Mean values ± SE of the number of total- and coffee-molecules smelled during the GC-O experiments by subjects, according to their TDI olfactory status. * Indicates significant differences between individuals with normosmia or hyposmia (*p* < 0.0001; Fisher’s LSD test subsequent to one-way ANOVA). (**B**) Mean values ± SE of the number of total- and coffee-molecules smelled during the GC-O experiments by females and males, according to their TDI olfactory status. Different letters indicate significant differences between individuals with normosmia or hyposmia (females: a-a_i_; males: b-b_i_; *p* < 0.01; Fisher’s LSD test subsequent to one-way ANOVA). * Indicates significant differences between females and males within the same TDI olfactory status (*p* ≤ 0.012; Fisher’s LSD test subsequent to one-way ANOVA).

Figure 1A shows the mean values ± SE of the number of odor-active compounds for individuals with normosmia or hyposmia. One-way ANOVA revealed that normosmic individuals detected a higher number of total-molecules (F (1,65) = 40.15; *p* < 0.0001) and coffee-molecules (F (1,65) = 32.54; *p* < 0.0001) than hyposmic ones. Figure 1B shows the same data according to sex. Post hoc comparisons, subsequent to two-way ANOVA (F (1,63) = 1.2162; *p* = 0.27), showed that normosmic individuals perceive a larger number of both total- and coffee-molecules, even when females (*p* < 0.0001; Fisher’s LSD test) and males (*p* ≤ 0.0077; Fisher’s LSD test) are considered separately. Furthermore, among normosmic individuals, the results show that females perceive a larger number of both total- (*p* = 0.0087; Fisher’s LSD test) and coffee-molecules (*p* = 0.0116; Fisher’s LSD test) than males.

**Figure 2:**
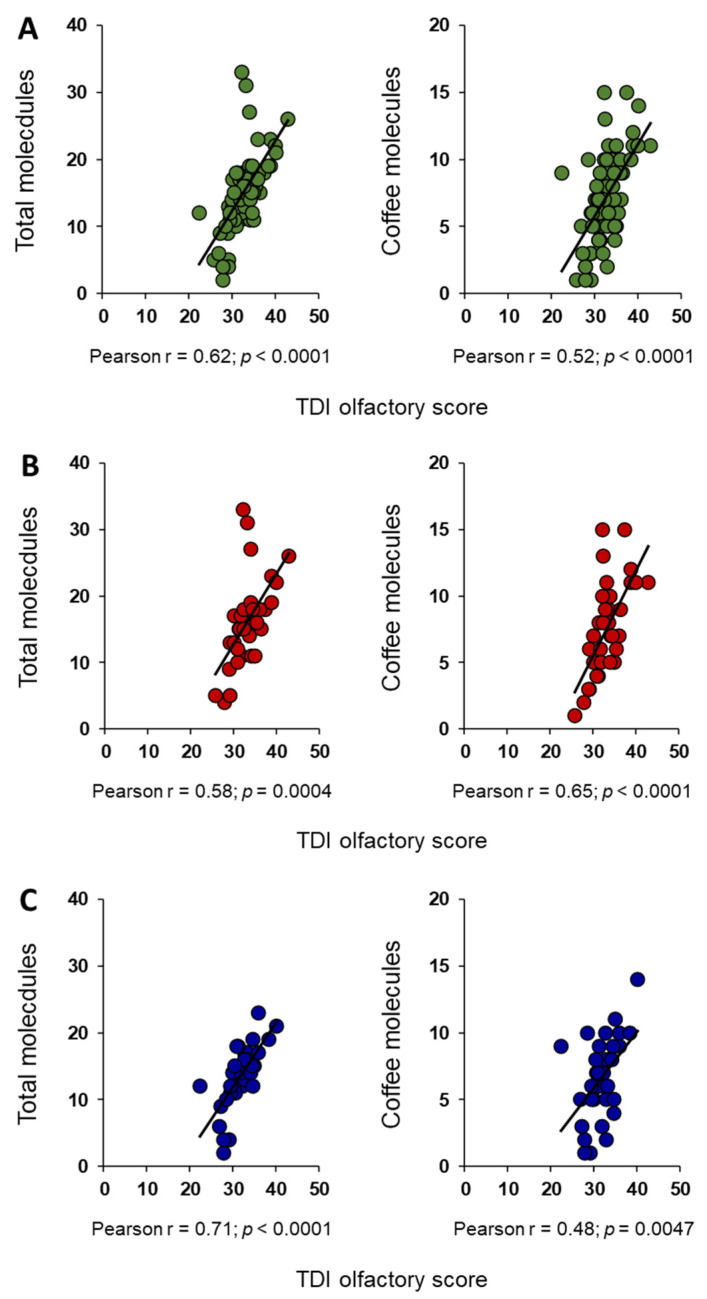
Relationship between TDI olfactory score and ability to perceive single molecules. Correlation analyses between TDI olfactory score and the number of total- and coffee-molecules smelled by subjects of both sexes together (**A**), only females (**B**) or only males (**C**).

The results of Pearson’s correlation test, shown in Figure 2A, indicate that the TDI olfactory score was positively correlated with both the number of total-molecules (Pearson’s r = 0.62, *p* < 0.0001) and that of coffee-molecules smelled by each subject (Pearson’s r = 0.52, *p* < 0.0001). Positive correlations between TDI olfactory score and the number of both total and coffee odor-active compounds were also found when females (Total: Pearson’s r = 0.58, *p* = 0.0004; Coffee: Pearson’s r = 0.65, *p* < 0.0001; Figure 2B) and males (Total: Pearson’s r = 0.71, *p* < 0.0001; Coffee: Pearson’s r = 0.48, *p* = 0.0047; Figure 2C) were considered separately.

**Figure 3:**
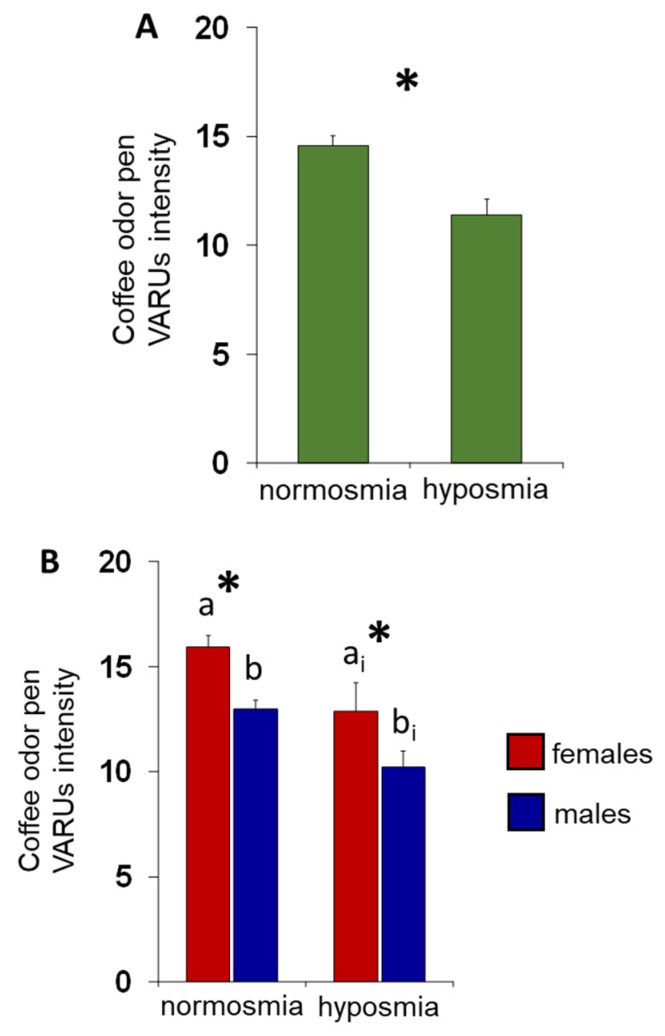
Relationship between effect of the TDI olfactory status on the intensity perceived for coffee-odor pen. (**A**) Mean values ± SE of the intensity perceived for coffee-odor pen by subjects according to their TDI olfactory status. * Indicates significant differences between individuals with normosmia or hyposmia (*p* = 0.0002; Fisher’s LSD test subsequent to one-way ANOVA). (**B**) Mean values ± SE of the intensity perceived for coffee-odor pen by each female and male separately, according to their TDI olfactory status. Different letters indicate significant differences between individuals with normosmia or hyposmia (females: a-a_i_; males: b-b_i_; *p* < 0.01; Fisher’s LSD test subsequent to one-way ANOVA). * Indicates significant differences between females and males within the same TDI olfactory status (*p* ≤ 0.027; Fisher’s LSD test subsequent to one-way ANOVA).

The mean values ± SE of the intensity perceived for the coffee-odor pen by panelists classified by their TDI olfactory status are shown in Figure 3A. One-way ANOVA revealed that the intensity perceived by normosmic individuals was significantly higher than that of hyposmic individuals (F (1,65) = 10.40, *p* = 0.0019). Figure 3B shows the same data according to sex. Post hoc analyses subsequent to two-way ANOVA (F (1,63) = 0.03; *p* = 0.87) highlighted that both normosmic females and males reported perceiving the coffee-odor pen with higher intensity than females (*p* = 0.0057; Fisher’s LSD test) and males (*p* = 0.0072; Fisher’s LSD test) with hyposmia. In addition, females perceived the coffee-odor pen more intensely than males, both among normosmic (*p* = 0.001; Fisher’s LSD test) and hyposmic (*p* = 0.0267; Fisher’s LSD test) individuals.

**Figure 4:**
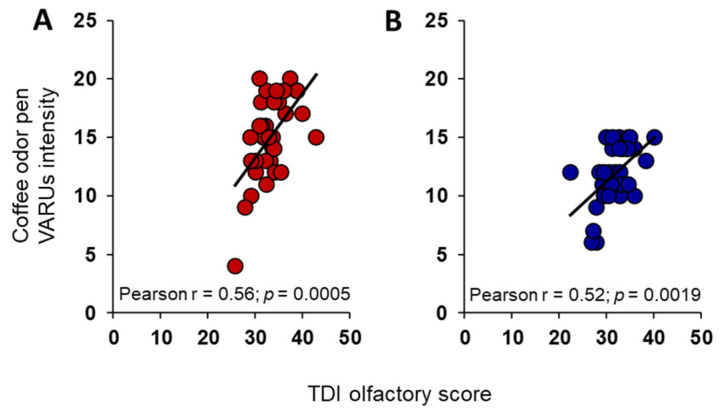
Relationship between TDI olfactory status and intensity perceived for coffee-odor pen. Correlation analyses between the intensity perceived for coffee-odor pen by each female (**A**) and male (**B**).

Pearson’s correlation analyses also revealed that the coffee-odor pen intensity reported by each individual was positively correlated with their TDI olfactory score, in both females (Pearson’s r = 0.56, *p* = 0.0005; Figure 4A) and males (Pearson’s r = 0.52, *p* = 0.0019; Figure 4B).

**Figure 5:**
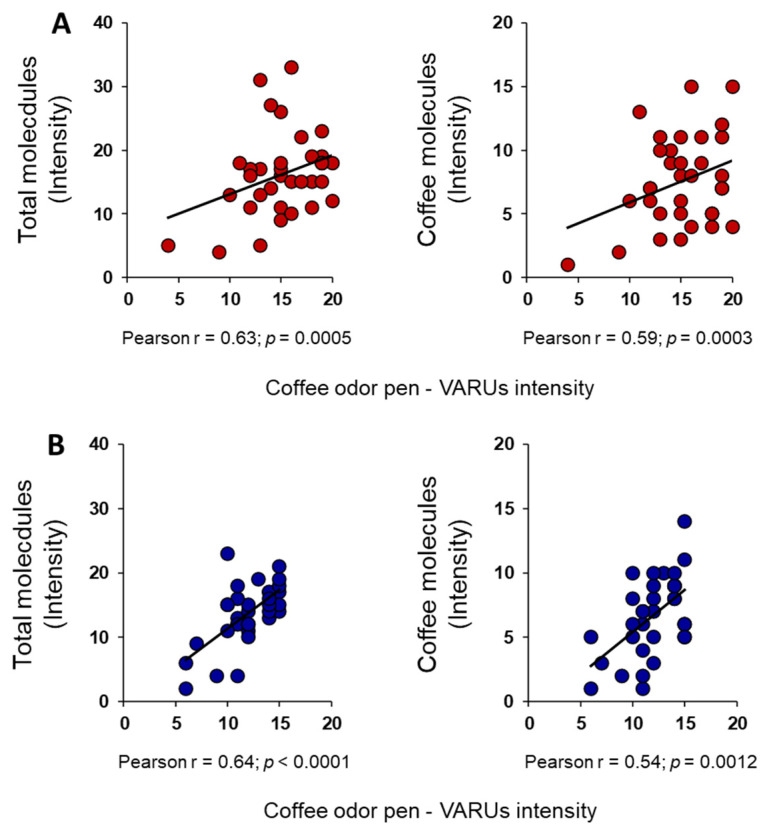
Relationship between the intensity perceived for coffee-odor pen and number of single molecules. Correlation analyses between the number of total- and coffee-molecules smelled and the intensity perceived for the coffee-odor pen by each female (**A**) and male (**B**).

In addition, we found a significant positive correlation between coffee-odor pen intensity and number of total- and coffee-molecules perceived by each female (Total: Pearson’s r = 0.63, *p* = 0.0005; Coffee: Pearson’s r = 0.59, *p* = 0.0003; Figure 5A) and male (Total: Pearson’s r = 0.64, *p* < 0.0001; Coffee: Pearson’s r = 0.54, *p* = 0.0012; Figure 5B).

## 4. Discussion

The olfactory system provides information both on the composition of the external environment—signaling the presence of dangers, influencing interindividual relationships and eating behavior—and on the composition of the internal environment, acting as a metabolic sensor [1,2,3,4,5,6,7,11,12,110,111]. Although the number of genes coding for functional olfactory receptors is approximately 350, it is known that the human nose, through a combinatorial code, is capable of perceiving and recognizing thousands of molecules [112,113,114,115,116,117,118,119]. Considering that the odor of foods and drinks is generally represented by a combination of several chemical molecules, that the sensorially active molecules are those that contribute most to determining the odor of the mixture and that the odor-active compounds differ between individuals, this may explain why the intensity and pleasantness with which a complex odor is perceived can vary greatly between individuals and be extremely personal [33,35,39,41,90].

Based on these considerations, as a first objective of this study we evaluated the ability of individuals to perceive single molecules as they are separated and eluted from a chromatographic column and conveyed, via an olfactometric port, to the nose of participants. The results obtained with the GC-O experiments show that the number of molecules, both total or having the coffee-odor, smelled by normosmic participants is significantly higher than the number of molecules perceived by the hyposmic participants, both when females and males are considered together and separately. On the basis of the positive correlations found between the number of molecules perceived and the TDI olfactory score obtained by each female and male, these results confirm, on the one hand, the close relationship between the ability to perceive single molecules and the olfactory function of individuals, and, on the other hand, that this also applies to females and males separately [39,41]. In fact, considering that one of the topics still-debated is whether the olfactory function of females differs from that of males, the main objective of this study was to evaluate the presence of differences, both qualitative (type of molecules perceived) and quantitative (number of molecules perceived), in the ability of females and males to perceive the single molecules that make up a complex mixture. The results show that females perceive a larger number of both total- and coffee-molecules than males, which is in agreement with previous studies on sex-related differences in olfactory function, which report that females perform better than males [52,63,120]. The reasons for this difference may be linked to cognitive, social and/or genetic factors. Previous studies have shown that females perform better than males in episodic olfactory memory, appear to be more interested in olfactory stimuli and are more familiar with odors [54,120,121,122]; in accordance, the frequency detection method used in this study to evaluate the ability to perceive single molecules, validated when using inexperienced evaluators, and especially when the mixture is unknown, requires good concentration and an ability to recall information from olfactory memory [92,93,123,124]. Regarding genetic factors, recent studies have shown that the expression and functionality of Kv1.3 channels, abundantly expressed in the olfactory epithelium and olfactory bulb, can play an important role in influencing the olfactory function of individuals [52,125,126,127]. In particular, one major T allele may protect females from olfactory dysfunction, while males need two T alleles for an olfactory performance that is comparable to that of females [52].

Another interesting aspect highlighted by our results is that females differ from males not only in the number of molecules perceived, but also in the type. In fact, we found that for six of the eighteen coffee-molecules, among participants who correctly identified them, the number of females was significantly higher than that of males. Even among the total-molecules, i.e., those belonging to the mixture but not classified as coffee odorants, we found sex-related differences among the participants who smelled them. In detail, two molecules were perceived by a larger number of females, while three others were perceived by a larger number of males. Finally, our results showed that females reported perceiving the coffee-odor pen with a significantly higher intensity than males, although, for both sexes, the correlation analyses showed that the intensity perceived by each participant was significantly correlated with their TDI olfactory score. This result can be partly explained by the fact that females not only perceive a larger number of both total- and coffee-molecules, but among the participants who smelled some of the coffee-molecules, the larger number is represented by females. Taken together, these findings support previous studies that highlight a better olfactory performance of females compared to males, but also the idea that each individual has their own sensory idea of a complex odor, despite everyone defining it as the same thing.

In this study, the olfactory function of individuals was assigned by means of the TDI olfactory score obtained from each participant. Since the TDI score is given by the sum of the score obtained with the olfactory threshold test (T-test), discrimination (D-test) and identification (I-test) of odors, the state of hyposmia can be determined by the reduced ability to perceive and/or discriminate and/or identify odors. We have previously found that in healthy subjects the main determinant of the TDI score is T-score, followed by D-score and finally by I-score [59]. This aspect has particular importance if we consider that, in GC-O experiments, the ability to perceive and discriminate odors is fundamental. First, the lower the olfactory threshold, the larger the number of molecules that can be perceived; in fact, even molecules that are subthreshold for individuals with reduced olfactory perception could instead be suprathreshold for those who show a better olfactory threshold. Second, a reduced ability to discriminate odors could cause similar odors to be perceived as the same, thus reducing the number of odor-active compounds. In agreement with this, a previous study highlighted that the ability to perceive single molecules was associated with the threshold olfactory score obtained by participants [41]. Furthermore, the differences found between sexes are also in agreement with recent findings on the differences between the TDI olfactory score of females and males, due to differences between the olfactory threshold and discrimination scores [52].

## 5. Conclusions

By considering that the olfactory function plays an important role in food choices, the ability to perceive odors, both simple and complex, as well as the type of molecules that are perceived, it is of particular importance for the state of health of individuals. In fact, the number, type and intensity with which the molecules are perceived can influence the quality and quantity of a meal. Consequently, if this is unbalanced towards foods with high energy content, we observe an increase in body weight and the appearance of unhealthy conditions such as dysglycemia and/or dyslipidemia, just to name a couple. A good olfactory function, on the other hand, favors both less abundant meals and meals rich in healthier foods such as fruit and vegetables.

The considerations emerging from the results of this study provide the basis for evaluating the following factors in future studies with a larger sample: (a) further differences, especially qualitative, between females and males in their ability to perceive single molecules as they are eluted from a chromatographic column; (b) the relationships between number and/or type of molecules perceived, and the olfactory threshold and discrimination in females and males separately and (c) the role of polymorphisms of some genes involved in the olfactory function of individuals.

## Figures and Tables

**Table 1:** GC-O analysis of volatile compounds found in the headspace of roasted coffee beans: odor-active compounds and odor descriptions by subjects.

N.	Odor-Active Compound	Odor Description	df (F-M)
1	Octane, 3,5-dimethyl-	Woody, burnt, unknown	0-3
2	Oxalic acid, isobutyl nonyl ester	Burnt, unknown	2-1
3	Toluene	Coffee, smoked, solvent, roasted, fruit	14-6
4	β-Pinene	Sweet, floral, vanilla, herbs, incense, sulfur, pungent	6-6
5	Ethylbenzene	Petrol	0-1
6	p-Xylene	Vanilla, medicinal, floral, gas, pungent	4-5
7	Oxalic acid, isobutyl pentyl ester	Floral, fruity, vanilla, sweet	5-3
8	Pyridine *	Coffee, smoked, roasted, cheese	14-3
9	D-Limonene *	Sweet, sour, citrus	1-6
10	Furan, 2-pentyl- *	Smoked, plastic, herbs	3-3
11	Pyrazine, methyl- *	Coffee, nutty, roasted, smoke, caramellic	3-5
12	Acetoin	Coffee, sweet, roasted, parfum, woody, caramellic	10-10
13	2-Propanone, 1-hydroxy-	Sweet, pungent, fish, solvent, wet, feet, medicinal	7-15
14	Pyrazine, 2,5-dimethyl- *	Coffee, citrus, medicinal, sweet, cocoa, shoes	7-10
15	Pyrazine, ethyl- *	Coffee, nutty, egg, pungent, shoes	4-2
16	Pyrazine, 2,3-dimethyl- *	Coffee, burnt, caramellic, fruity	4-3
17	DL-2,3-Butanediol *	Sweet, caramellic, rose, wet	2-4
18	Vinyl butyrate	Floral, parfum, bitter, solvent, pungent, plastic	5-4
19	Hex-4-yn-3-one, 2,2-dimethyl-	Sweet, solvent, pungent	2-3
20	Pyrazine, 2-ethyl-6-methyl- *	Coffee, sweet, smoked, medicinal, solvent, parfum, roasted, balsamic, fruit	19-25
21	Pyrazine, 2-ethyl-3-methyl- *	Coffee, cocoa, solvent, bitter, nutty, roasted, burnt, medicinal, solvent, herbs	24-22
22	Pyrazine, 2-(n-propyl)- *	Green, musty, woody, earthy, wet, herbs, floral, fruit	18-16
23	Pyrazine, 2,6-diethyl- *	Coffee, roasted, earthy, musty, burnt, mushrooms, vegetable	23-21
24	Pyrazine, 3-ethyl-2,5-dimethyl- *	Coffee, nutty, roasted, floral, bitter, woody, solvent, wet	18-16
25	2-Propanone, 1-(acetyloxy)-	Pungent, parfum, wet	6-3
26	Pyrazine, 2-ethyl-3,5-dimethyl- *	Coffee, musty, roasted, wet, herbs, musty	17-19
27	Furfural *	Coffee, sweet, solvent, floral, pungent	15-6
28	Pyrazine, tetramethyl-	Coffee, roasted, burnt, vanilla, bitter, solvent	13-13
29	Pyrazine, 3,5-diethyl-2-methyl- *	Floral, musty, wet, solvent, fresh	11-20
30	Pyrazine, 2-ethenyl-5-methyl-	Coffee, nutty, bitter, plastic, earthy, musty	15-11
31	Furan, 2-acetyl- *	Parfum	2-0
32	2,3-Pentanedione *	Floral, earthy, sweat, musk, cheese, pungent, woody	26-24
33	2-Butanone, 1-(acetyloxy)-	----------	0-0
34	2-Furanmethanol, acetate *	Roasted, fruit, herb, woody, coffee, vegetable, fish	22-12
35	Pyrazine, 2-methyl-6-(2-propenyl)-	Pungent, sour, bitter, herbs, spicy	8-1
36	2-Cyclopenten-1-one, 2,3-dimethyl-	Sweet, floral, lavender	3-1
37	Acetic acid, diethyl- *	Roasted, solvent, rotten, musty, wet earth, coffee	20-13
38	Pentanoic acid, 4-oxo-, methyl ester	Sweet, nutty	5-2
39	2-Furancarboxaldehyde, 5-methyl- *	Coffee, sweet, parfum, solvent	5-4
40	2-Furanmethanol, propanoate *	Coffee, pungent, floral, musty, herb, sweet, burnt, vegetable	14-9
41	Furan, 2,2′-methylenebis- *	Coffee, nutty, popcorn, roasted, fish, sour, plastic, smoke	22-12
42	2-Furanmethanol *	Coffee, smoke, popcorn, nutty, roasted, sweet	22-12
43	Butanoic acid, 3-methyl- *	Cheese, smoke, stinky feet, acidic, fruity, putrid	14-19
44	Furan, 2-(2-furanylmethyl)-5-methyl- *	Nutty, plastic, unknown	1-2
45	Pyrazine, 2-acetyl-6-methyl	Putrid, musty, cheese, medicinal	4-6
46	4(H)-Pyridine, N-acetyl- *	Shoes, wet, sweat, plastic, cheese	9-7
47	Octaethylene glycol monododecyl ether	Sweat, acidic	2-2
48	2-Hexadecanol	Cheese, musty, putrid, plastic, shoes, burnt	24-23
49	N-Furfurylpyrrole *	Solvent, cheese, musty, coffee, caramellic, smoked	16-20
50	2-Acetylpyrrole *	Coffee, roasted, almond, sweet, burnt, parfum, fresh, popcorn	25-15

Odor-active compounds: list of compounds eluted by the chromatographic column during GC-O experiments and smelled by at least two subjects who participated in the study. Odor description: specific description that each subject gave of the odor smelled during the GC-O experiment. df = detection frequency; number of females and males who smelled the compound. Volatile compounds described in the literature as molecules smelling of coffee are listed in red print. Asterisk indicates molecules commonly found in the headspace of roasted coffee beans [100,101,102,103,104,105,106,107,108].

**Table 2:** GC-O analysis: sex-related differences for some odor-active compounds.

Molecule	Perception Ability	F *n* (%)	M *n* (%)	*p*-Value
Toluene	Yes	14	6	0.039
	No	20	27	
Pyridine	Yes	14	3	0.003
	No	20	30	
D-limonene	Yes	1	6	0.041
	No	33	27	
2-Propanone, 1-hydroxy-	Yes	7	15	0.030
	No	27	18	
Furfural	Yes	15	6	0.022
	No	19	27	
Pyrazine, 3,5-diethyl-2-methyl-	Yes	11	20	0.020
	No	23	13	
2-Furanmethanol, acetate	Yes	22	12	0.020
	No	12	21	
Pyrazine, 2-methyl-6-(2-propenyl)-	Yes	8	1	0.014
	No	26	32	
Furan,2,2-methylenebis-	Yes	22	12	0.020
	No	12	21	
2-Furanmethanol	Yes	22	12	0.020
	No	12	21	
2-Acetylpyrrole	Yes	25	15	0.019
	No	9	18	

Different distribution between females (F) and males (M) in their ability to perceive some molecules from the headspace of roasted coffee beans. *p*-value derived from Fisher’s Exact Test. Females (n = 34), males (n = 33). Volatile compounds described in the literature as molecules smelling of coffee are listed in red print.

## Data Availability

The original contributions presented in the study are included in the article, further inquiries can be directed to the corresponding author.
